# Prognostic role of neutrophil-to-lymphocyte ratio in aortic disease: a meta-analysis of observational studies

**DOI:** 10.1186/s13019-020-01263-3

**Published:** 2020-08-10

**Authors:** Yan Xu, Haiyang Fang, Zhiqiang Qiu, Xiaoshu Cheng

**Affiliations:** 1 Department of Cardiovascular Medicine, Institute of Cardiovascular disease, Second Affiliated Hospital of Nanchang University, Nan Chang, Jiang Xi 330006 PR China; 2 Department of Orthopedics, Second Affiliated Hospital of Nanchang University, Nanchang, Jiangxi China

**Keywords:** Neutrophil-to-lymphocyte ratio, Aortic disease, Aortic aneurysm, Aortic dissection, Meta-analysis

## Abstract

**Objective:**

Recent studies have reported that neutrophil-to-lymphocyte ratio (NLR) is associated with cardiovascular disease. The aim of the present study was to investigate the prognostic value of NLR in aortic disease.

**Methods:**

We systematically searched electronic databases (Cochrane, PubMed, Elsevier, Medline, and Embase) from their inception to March 2020. Observational studies that evaluated the relationship between NLR and aortic disease were eligible for critical appraisal. Data were extracted from applicable articles, risk ratio (RR), weighted mean differences (MD) and 95% confidence intervals (CI) were calculated by RevMan 5.3, and statistical heterogeneity was assessed by the I^2^ statistic.

**Results:**

Fourteen studies enrolling 4066 individuals were included in the meta-analysis. Compared with the control group, NLR was significantly higher in the aortic disease group (MD 3.44, 95%CI: 0.81–6.07, *P* = 0.01, I^2^ = 99%). The NLR was also significantly higher in non-survivors with aortic disease, compared to the survivors (MD 4.62, 95%CI: 2.75–6.50, *P* < 0.00001, I^2^ = 60%). Compared with the aortic disease patients with a low NLR, mortality was significantly higher in those with a high NLR (RR 2.63, 95%CI: 1.79–3.86, *P* < 0.00001, I^2^ = 67%).

**Conclusion:**

Based on current evidence, an elevated NLR was associated with aortic disease and in-hospital mortality. Raised NLR also demonstrated a significantly increased the risk of mortality after surgical repair in aortic disease patients. NLR may be a good prognostic biomarker in aortic disease and deserve further research in this area.

## Introduction

Aortic disease is common and consists of pathologies that are both congenital and acquired. Aortic aneurysm and aortic dissection are the most frequent types of aortic disease which have high complication rates and carry a high risk of mortality [1, 2]. Literature has shown that inflammation of the aortic wall is considered to be the principal causes of aortic disease. It has been found that inflammatory biomarkers such as macrophages, C-reactive protein, neutrophils, lymphocytes are elevated in aortic disease [3–5].

Complete blood count parameters are widely known markers of systemic inflammation and have been associated with various cardiovascular diseases [6, 7]. Neutrophils secret various enzymes and mediators to participate in inflammation. High neutrophil counts are highly susceptible to inflammation not only in infective disease, but also in cardiovascular diseases. Low lymphocyte counts reflect the inflammation that is associated with adverse outcomes in patients with cardiovascular diseases [8]. Therefore, the neutrophil to lymphocyte ratio (NLR) is easy to obtain, inexpensive and widely available that has been suggested as a new indicator of inflammation and a predictor of clinical outcomes in cardiovascular disease, in addition to traditional markers [9, 10].

Recently, the relationship between NLR and aortic disease has been investigated by several studies [11, 12]. Authors focus on the NLR level in aortic aneurysm and aortic dissection patients, and the predictive value of high NLR in mortality and other clinical outcomes [13–26]. Therefore, the current meta-analysis was performed to clarify the relationship between NLR and aortic disease.

## Methods

### Search strategy

This study was conducted according to the Preferred Reporting Items for Systematic Reviews and Meta-analyses (PRISMA). We performed a literature search by a comprehensive computer search of electronic databases (Cochrane, PubMed, Medline, Elsevier, and Embase) from their inception to March 2020. Medical subject headings (MeSH) and free-text words were used in the meta-analysis. The following search terms were used: “Neutrophil lymphocyte ratio (MeSH)”, “NLR (MeSH)”, “aortic disease (MeSH)”, “aortic aneurysm (MeSH)”, “aortic dissection (MeSH)”. When appropriate, Boolean operators (NOT, AND, OR) were used to widen or narrow the search range. The references, reviews and editorials in the retrieved articles were manually searched for relevant articles.

### Study selection

The inclusion criteria were listed as follows: (a) publications about observational studies focused on the relationship between NLR and aortic disease, (b) participants in the studies had aortic aneurysm or aortic dissection, (c) studies grouped with aortic disease and control, or high NLR and low NLR, and (d) The NLR in each group was presented as mean ± standard error (SD). Letters, reviews, case reports, animal studies, or non-English publications were excluded. All observational studies that met these requirements were considered eligible for the meta-analysis.

### Data extraction

The literature search, study selection, and data extraction were done independently by 2 investigators (Y. Xu and H. Fang). Any discrepancies were resolved by the coauthors (Z. Qiu and X. Cheng). The following data were collected: first author name, publication year, country, journal, sources of controls, sample size, cutoff values for NLR, and outcomes.

### Quality assessment

Newcastle-Ottawa scale (NOS) was used to assess the quality of the included studies by 2 investigators (Y. Xu and Z. Qiu) [27]. Studies included in the current meta-analysis was judged on three main dimensions: the selection of the study groups, the comparability of the study populations, and the determination of the exposure. The total score for a single study ranged from 0 to 9 stars. Studies whose scores less than 5 stars were categorized as low quality, scores 5–7 stars categorized as moderate quality, and scores more than 7 stars categorized as high quality. The mean score of studies in the meta-analysis was 7.8 stars. Another 11 items were used for assessing Kalkan ME’s study [26], which was a case-sectional study and recommend by AHRQ (Agency for Healthcare Research and Quality), the score is 10.

### Data synthesis and meta-analysis

The meta-analysis and statistical analyses were performed by Review Manager (RevMan) version 5.3 (The Nordic Cochrane Centre, Rigshospitalet, Copenhagen, Denmark). Weighted mean differences (MD), 95% confidence intervals (CI), and risk ratio (RR) were used to evaluate the association between NLR and aortic disease. The Mantel-Haenszel method for fixed effects and the DerSimonian-Laird method for random effects were used to estimate MD, RR. The heterogeneity among studies was tested by Cochran’s Q statistic and I^2^ test. A *P* < 0.1 and I^2^ test value > 50% indicated substantial heterogeneity; in that case, the summary estimate was analyzed by the random effects model. Otherwise, the fixed effects model was used. Statistical significance was set at a *P* < 0.05 (two-tailed).

## Results

### General characteristic of studies included in the meta-analysis

**Fig. 1:**
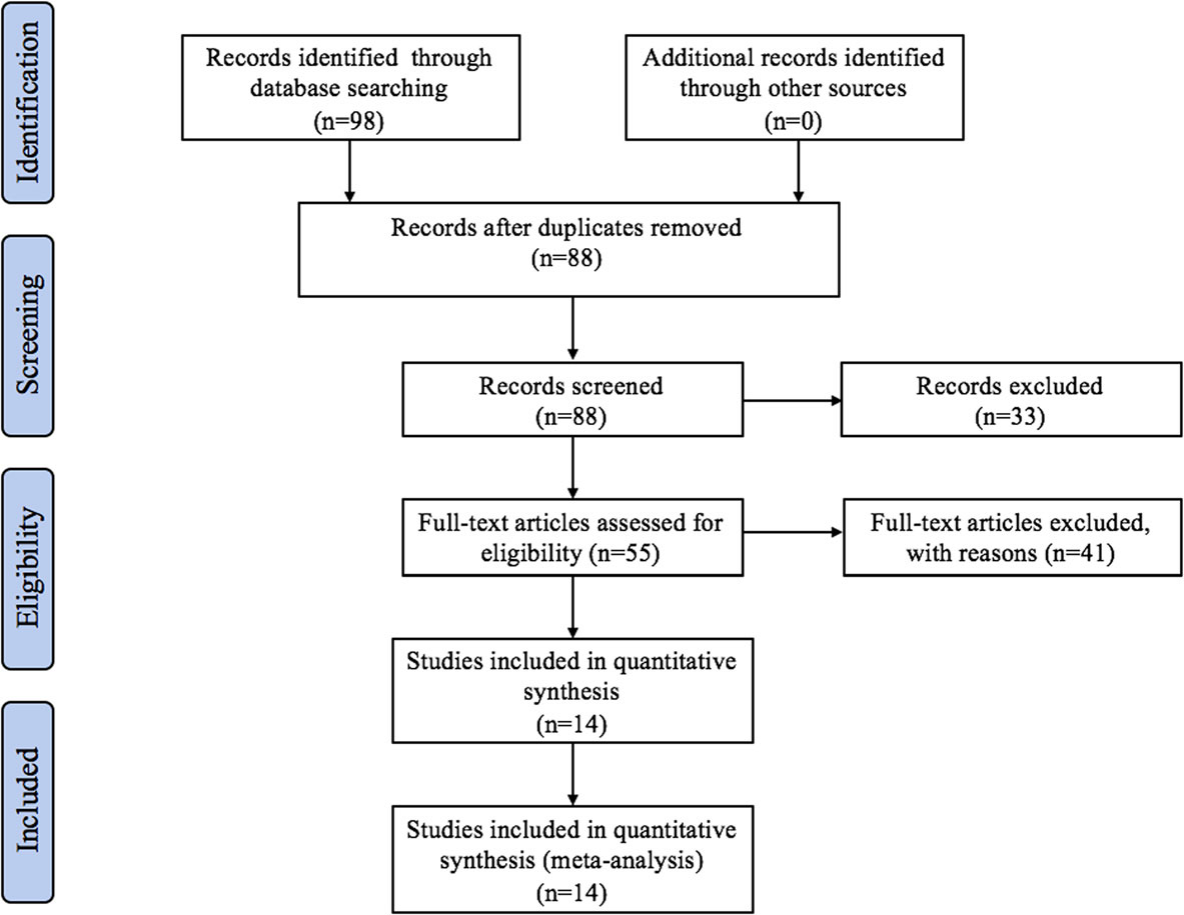
PRISM flowchart for search strategy and selection of eligible studies

The initial search produced 98 results, of which 23 records met the general inclusion criteria and were reviewed for strict screening the titles and abstracts, the PRISMA study flowchart was shown in Fig. 1. After reviewing the full-text articles, the remaining 14 observational studies were included in current meta-analysis [13–26].

The patient demographics and basic characteristics of the included studies were shown in Table 1. Overall, these studies included 4066 patients. Four studies evaluated the NLR value in aortic disease (*n* = 868). Six studies investigated the NLR value in survivor and non-survivor patients with aortic disease (*n* = 600). Seven studies estimated the relationship between high or low NLR and mortality in aortic disease (*n* = 2963). The cutoff values of NLR for dichotomization ranged from 3.5 to 9.7024. The worse outcomes included in-hospital mortality and long-term mortality, follow-up period ranged from 30 days to 10 years. Among these 14 studies, 13 were cohort studies, and 1 was a case-sectional study.

**Table 1:** General characteristics of studies included in meta-analysis

References	Time	Study design	Country	Sample size	Mean age (year)	Gender, M/F	Patients	Experiment group	Control group	NOS (stars/ scores)
Ikenaga H [13]	2014	Case-control	Japan	172	70.2 ± 9.7	130/42	Aortic aneurysm	Aortic aneurysm group	Control group	6
King AH [14]	2020	Retrospective cohort	USA	108	75.5 ± 8.5	7830	Aortic aneurysm	High NLR	Low NLR	9
Vuruskan E [15]	2016	Case-control	Turkey	219	70.2 ± 11.8	155/64	Aortic aneurysm	Aortic aneurysm group	Control group	7
Oz K [16]	2017	Retrospective cohort	Turkey	57	54.6 ± 10.3	9/48	Aortic dissection	deceased	survived	8
Onuk T [17]	2015	Retrospective cohort	Turkey	200	58 ± 12.7	133/67	Aortic dissection	deceased	survived	8
Bedel C [18]	2020	Retrospective cohort	Turkey	96	63.7 ± 13.6	78/18	Aortic dissection	deceased	survived	8
Karakoyun S [19]	2015	Retrospective cohort	Turkey	35	55.9 ± 7.6	28/9	Aortic dissection	deceased	survived	8
Lafci G [20]	2014	Retrospective cohort	Turkey	104	55.3 ± 13.5	76/28	Aortic dissection	deceased	survived	8
Appleton ND [21]	2014	Retrospective cohort	United Kingdom	350	72.9 ± 7.9	175/75	Aortic aneurysm	High NLR	Low NLR	9
Aurelian SV [15]	2019	Retrospective cohort	France	255	68.3 ± 7.6	230/25	Aortic aneurysm	High NLR	Low NLR	8
Bath J [23]	2019	Retrospective cohort	USA	1908	72.2	1458/450	Aortic aneurysm	High NLR	Low NLR	7
Kordzadeh A [24]	2015	Retrospective cohort	United Kingdom	80	75	66/14	Aortic aneurysm	High NLR	Low NLR	8
Lareyre F [25]	2018	Retrospective cohort	France	75	71	57/16	Aortic aneurysm	High NLR	Low NLR	8
Kalkan ME [26]	2015	cross-sectional study	Turkey	184	53.1 ± 11.4	134/50	Aortic dissection	High NLR	Low NLR	10

*M* Male, *F* Female, *NOS* Newcastle-Ottawa Scale, *NLR* Neutrophil-to-lymphocyte ratio

### The level of NLR in aortic disease

**Fig. 2:**
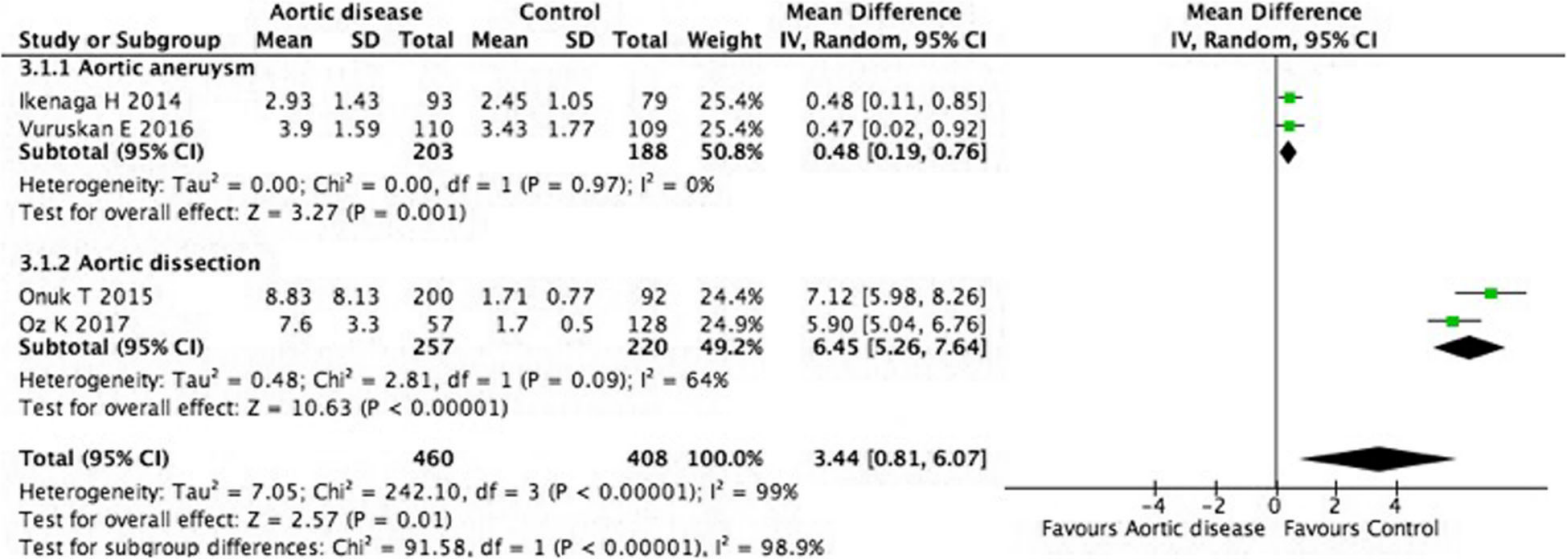
The level of Neutrophil-to-lymphocyte ratio in aortic disease

The relationship between the NLR value and aortic disease was reported in four studies that included 460 aortic disease patients and 408 controls [13, 15–17]. The patients with aortic aneurysm in two studies (*n* = 391), and with aortic dissection in other two studies (*n* = 477). Significant heterogeneity was found between the two groups (I^2^ = 99%) (Fig. 2). Analysis of the overall effect on NLR revealed a significant difference in NLR between individuals in the aortic disease and control group (MD 3.44, 95%CI: 0.81–6.07, *P* = 0.01). A subgroup analysis was conducted for patients from different aortic disease. The results showed NLR value was significantly higher in patients with aortic aneurysm and aortic dissection than individuals in the control group (MD 0.48, 95%CI: 0.19–0.76, *P* = 0.001, I^2^ = 0%; MD 6.45, 95%CI: 5.26–7.64, *P* < 0.00001, I^2^ = 64%). Compared with individuals in the control group, the NLR was significantly higher in aortic disease patients.

### The relationship between NLR and mortality in aortic disease

**Fig. 3:**
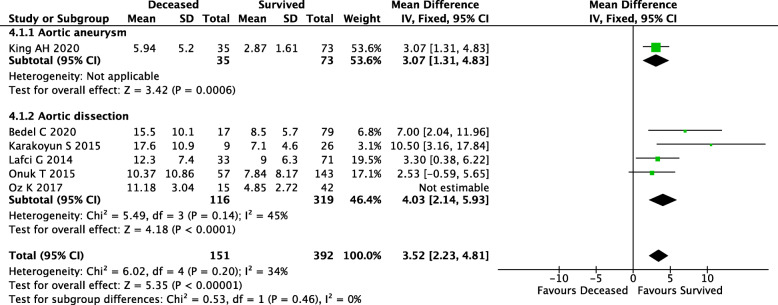
The relationship between Neutrophil-to-lymphocyte ratio and mortality in aortic disease

Researches have shown NLR is a predictive marker of mortality in aortic disease. Six studies investigated the NLR level in survivor and non-survivor patients with aortic disease [14, 16–20]. One hundred sixty-sixdeceased patients, and 434 survived patients were included in meta-analysis. The patients with aortic aneurysm in one study, and with aortic dissection in other five studies. Analysis of the overall effect revealed NLR value was significantly higher in non-survivor patients with aortic disease (MD 4.62, 95%CI: 2.75–6.5, *P* < 0.00001, I^2^ = 60%) (Fig. 3). We also performed a subgroup analysis according to different aortic disease. The results showed NLR was significantly higher in non-survivor patients than those in survivor patients (MD 5.15, 95%CI: 2.94–7.35, *P* < 0.00001, I^2^ = 53%).

### Prognostic value of high NLR for mortality in aortic disease

**Fig. 4:**
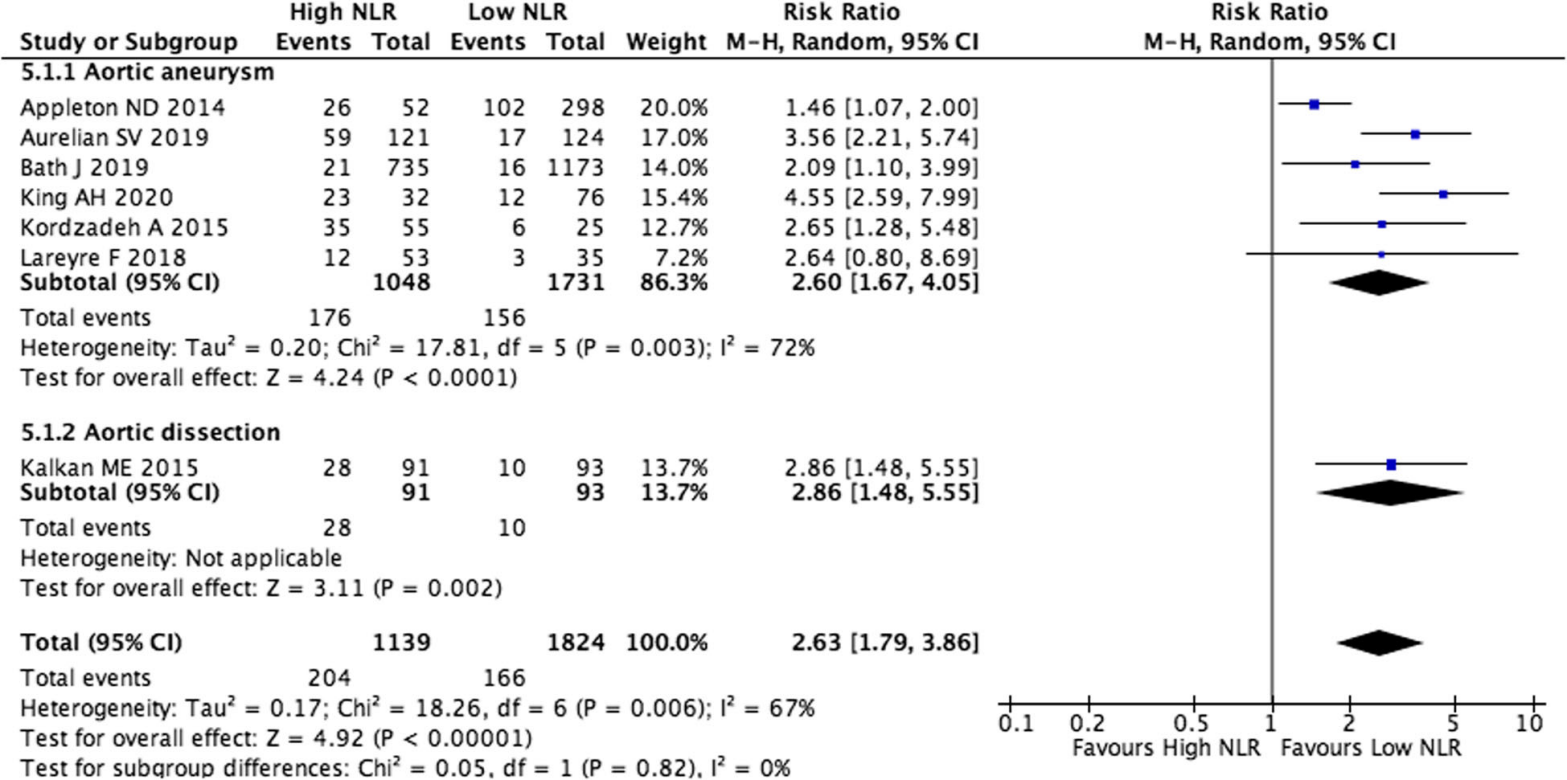
Prognostic value of high NLR for mortality in aortic disease

Seven studies also investigated the prognostic value of high NLR for mortality in aortic disease, the outcome was mortality [14, 21–26]. One thousand one hundred thirty-nine aortic disease patients in the high NLR group, and 1824 aortic disease patients in the low NLR group were included in meta-analysis (Fig. 4). The mortality rate in high NLR group of aortic disease patients was 17.91% (*n* = 204/1139), and in low NLR group was 9.1% (*n* = 166/1824). The patients with aortic aneurysm who underwent surgical repair in six studies, and with aortic dissection who underwent surgical repair in the other study. Analysis of the overall effect showed that the mortality in high NLR group of aortic disease patients was 2.63 times higher than that in low NLR group (RR 2.63, 95%CI: 1.79–3.86, *P* < 0.00001, I^2^ = 67%). Result of subgroup analysis also showed that NLR greater than the cutoff value was associated with higher mortality in aortic aneurysm patients (RR 2.60, 95%CI: 1.67–4.05, *P* < 0.0001, I^2^ = 72%). Overall, aortic disease patients with a high NLR had significantly higher mortality after surgical repair than those with a low NLR.

### Sensitivity analysis and publication bias

The heterogeneity among studies reporting on the relationship between NLR and mortality changed to 34% after eliminating the study by Oz K et al. [16] (MD 3.52, 95%CI: 2.23–4.81, *P* < 0.00001) (Supplemental Fig. 1). The heterogeneity among studies reporting on prognostic value of high NLR for mortality changed to 0% after eliminating the study by Appleton ND et al. [21] (RR 3.06, 95%CI: 2.36–3.98, *P* < 0.00001) (Supplemental Fig. 2).

Funnel plot, Egger’s and Begg’s test are used to access publication bias in most meta-analysis. We did not assess publication bias in this meta-analysis, because the number of trials was low.

## Discussion

Accumulated evidence has shown that NLR is a simple and reliable marker for predicting worse outcomes in systematic inflammatory disease [28, 29]. In this meta-analysis, we evaluated the prognostic role of NLR in aortic disease from 14 observational studies. The results showed aortic disease patients and these patients who died on hospitalization had higher NLR. Raised NLR also demonstrated a significantly increased the risk of mortality after surgical repair in 2963 patients with aortic disease. According to our literature screening, this is the first meta-analysis to evaluate the link between NLR and aortic disease.

Inflammation represents a main pathophysiological feature contributing to the development of aortic aneurysm and aortic dissection [30, 31]. It is plausible that the damaged aorta, due to the inflammatory process within the wall and intima, may enlarge more easily and may be more prone to re-dissection and rupture [32, 33]. Vrsalovic M et al. conducted a systematic review and found elevated admission C-reactive protein levels indicated increased in-hospital and medium-term mortality in aortic disease [34]. The high white blood cell count level on admission was related to high in-hospital mortality in patients with aortic dissection [35]. Elevated NLR correlated well with other markers of inflammation, such as C-reactive protein, interleukin-6, tumor necrosis factor-alpha [36, 37]. NLR was first described in 1967 as a novel inflammatory biomarker that recently been linked to cardiovascular disease, such as heart failure, coronary artery disease (CAD) [38]. In the present study, we demonstrated the association between the NLR and aortic disease, this result was similar with others [14, 15, 17].

Several factors have been suggested to be associated with poor outcomes in aortic disease patients, such as older age, the large diameter of the descending aorta, and shock [39, 40]. However, few effective biomarkers predicting the prognosis of patients with aortic disease are currently available [40]. In our meta-analysis, we found that elevated NLR predicted worse outcomes in patients with aortic aneurysm and aortic dissection. Tan TP et al. conducted a meta-analysis to investigate the prognostic value of NLR in cardiovascular surgery. They found that raised NLR appeared to be associated with increased mortality and morbidity after cardiac and vascular surgery [10]. Elevated NLR was also associated with increasingly severe symptoms of peripheral artery disease in a graded response from claudication to tissue loss, and was independently associated with preoperatively and postoperatively and complications after lower extremity procedures [41].

C-reactive protein has been widely used in the clinical setting to assess inflammatory disorders. However, Wada H et al. found NLR was strongly associated with poor clinical outcomes in CAD patients with low C-reactive protein levels [42]. In the Rotterdam study, even with the addition of C-reactive protein in the multivariate model, an elevated NLR remained an independent predictor of all-cause mortality and cardiovascular mortality [43]. De jager CP et al. [44] also found NLR predict bacteremia better than conventional infection markers like C-reactive protein, white blood cell count and neutrophil in emergency department admission. NLR was a better predictor of weaning failure than leukocyte levels and C-reactive protein in patients receiving invasive mechanical ventilation [45]. Previous studies have reported that NLR is associated with malignant diseases [46]. In included studies of the meta-analysis, patients with relatively high NLR were excluded active inflammatory disease and known malignancy. Overall, NLR is a simple, widely applied, and inexpensive and easily accessible inflammatory marker in various cardiac and vascular diseases.

In our meta-analysis, we found the NLR level was significantly higher in aortic aneurysm and aortic dissection patients compared with controls, and there was a great disparity between the aortic aneurysm subgroup (MD 0.48) and the aortic dissection subgroup (MD 6.45). The patients with aortic aneurysm included in our meta-analysis were non-ruptured aortic aneurysm patients, the inflammatory response occurred in these patients was chronic and low-grade. However, the patients with aortic dissection patients were acute aortic dissection patients. Acute aortic dissection and ruptured aortic aneurysm were emergency and life-threatening diseases associated with severe mortality, a strong inflammatory response occurred in acute and ruptured status. The level of NLR was significantly higher in patients with ruptured aortic aneurysm compared with those with non-ruptured [15, 18]. The subgroup analysis results showed patients with aortic aneurysm and aortic dissection had higher NLR than the controls with moderate heterogeneity (I^2^ = 0%, I^2^ = 64%), but a significant heterogeneity was detected when the data were combined and analysed (I^2^ = 99%), the great disparity between two subgroups may be the sources of heterogeneity.

Our meta-analysis has several limitations. First, marked heterogeneity among studies was observed, the source of heterogeneity need to be explored. Second, NLR has been reported in a variety of different ways, and there has not been an established method for reporting NLR. Third, the cutoff value of NLR in included studies are different. The most of patients were men. Finally, all studies in the meta-analysis were observational. Large- scale RCT trials are necessary to further validate the current results.

## Conclusions

In conclusion, this meta-analysis showed that an elevated NLR was associated with aortic disease and in-hospital mortality. Raised NLR also demonstrated a significantly increased the risk of mortality after surgical repair in aortic disease patients. NLR may be a good prognostic biomarker in aortic disease and deserve further research in this area.

## Supplementary information

**Additional file 1.** Supplemental Figure 1 The sensitive analysis for the relationship between NLR and mortality.

**Additional file 2.** Supplemental Figure 2 The sensitive analysis for prognostic value of high NLR for mortality.

## Data Availability

All data generated or analysed during this study are included in this published article.

## Abbreviations

NLR: Neutrophil-to-lymphocyte ratio
RevMan: Review Manager
RR: Risk ratio
MD: Weighted mean differences
CI: Confidence intervals
SD: Standard error
NOS: Newcastle-Ottawa scale
AHRQ: Agency for Healthcare Research and Quality
CAD: Coronary artery disease
