# Supplementary figures and images for: Prognostic role of neutrophil-to-lymphocyte ratio in aortic disease: a meta-analysis of observational studies
Source: J Cardiothorac Surg. 2020 Aug 10;15:215. doi: 10.1186/s13019-020-01263-3 (PMC7419193; doi:10.1186/s13019-020-01263-3)

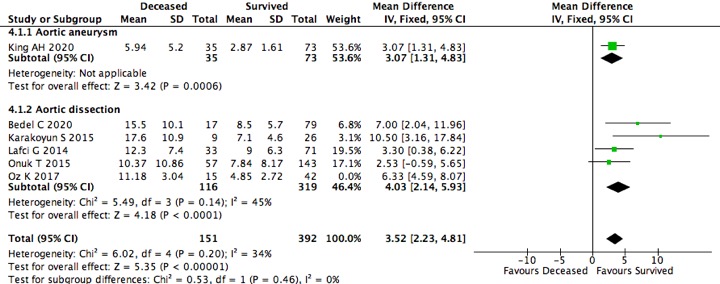

Supplement: Supplementary file 1 — Additional file 1. Supplemental Figure 1 The sensitive analysis for the relationship between NLR and mortality. [file 13019_2020_1263_MOESM1_ESM.jpg]

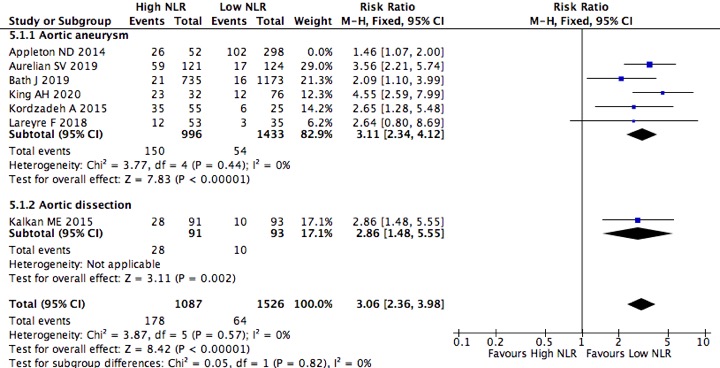

Supplement: Supplementary file 2 — Additional file 2. Supplemental Figure 2 The sensitive analysis for prognostic value of high NLR for mortality. [file 13019_2020_1263_MOESM2_ESM.jpg]
